# Advancements in brain-machine interfaces for application in the metaverse

**DOI:** 10.3389/fnins.2024.1383319

**Published:** 2024-06-11

**Authors:** Yang Liu, Ruibin Liu, Jinnian Ge, Yue Wang

**Affiliations:** ^1^Department of Ophthalmology, First Hospital of China Medical University, Shengyang, China; ^2^Department of Clinical Integration of Traditional Chinese and Western medicine, Liaoning University of Traditional Chinese Medicine, Shenyang, China; ^3^Department of General Surgery, Cancer Hospital of China Medical University, Liaoning Cancer Hospital & Institute, Shenyang, China; ^4^Department of General Surgery, First Hospital of China Medical University, Shengyang, China

**Keywords:** brain-machine interface, metaverse, human-computer interaction, neurosecurity, application

## Abstract

In recent years, with the shift of focus in metaverse research toward content exchange and social interaction, breaking through the current bottleneck of audio-visual media interaction has become an urgent issue. The use of brain-machine interfaces for sensory simulation is one of the proposed solutions. Currently, brain-machine interfaces have demonstrated irreplaceable potential as physiological signal acquisition tools in various fields within the metaverse. This study explores three application scenarios: generative art in the metaverse, serious gaming for healthcare in metaverse medicine, and brain-machine interface applications for facial expression synthesis in the virtual society of the metaverse. It investigates existing commercial products and patents (such as MindWave Mobile, GVS, and Galea), draws analogies with the development processes of network security and neurosecurity, bioethics and neuroethics, and discusses the challenges and potential issues that may arise when brain-machine interfaces mature and are widely applied. Furthermore, it looks ahead to the diverse possibilities of deep and varied applications of brain-machine interfaces in the metaverse in the future.

## 1 Introduction

The metaverse, a concept envisioning the Internet as a unified virtual world facilitated by virtual reality (VR) and augmented reality (AR) headsets, represents interconnected 3D virtual realms for social and economic interactions (Bhugaonkar et al., 2022). It is defined as a three-dimensional online environment where users, represented by avatars, engage in detached virtual interactions. The metaverse’s development is closely linked to advancements in VR technology and encompasses hardware, software, content, user interactions, virtual space realization, and diverse applications (Kye et al., 2021; Ritterbusch and Teichmann, 2023). While prior research focused on structural aspects, recent emphasis lies on content exchange and social interaction functionalities, enhancing interactive capabilities. Sensory simulation studies, including Brain-Machine Interfaces (BMIs), have gained importance for authentic real-world interaction simulation within the metaverse. Presently, there are no specific instances combining BMI sensory simulation with metaverse technology, though BMIs primarily serve as tools for physiological data acquisition within the metaverse (Wang et al., 2022). Examining the current status and challenges of BMI technology application within the metaverse provides insight into potential future developments of BMI sensory simulation technology.

## 2 Progress in the application of brain-machine interfaces in the metaverse

A Brain-Machine Interfaces (BMI) enables direct communication between the human brain and external devices without muscular control. BMIs decode brain signals to control devices, aiding individuals with disabilities, enhancing cognitive abilities, and potentially transforming human-computer interaction. In the medical field, BMIs benefit individuals with paralysis or neurological disorders by enabling control of assistive devices through brain activity detection methods like EEG and fMRI (Kuruvilla and Flink, 2003; Palmini, 2006). Advancements in BMI technology, including front-end interfaces, backend algorithms, wearable devices, and EEG, offer diverse applications such as the BMI speller and neural prostheses, improving quality of life for users. BMIs also show promise in gaming, allowing players to interact using motor imagery and SSVEP (Baumeister, 2000; Hariz et al., 2016). Non-invasive BMIs are preferred for EEG detection due to their ease of use and accuracy. BMI technology’s potential in the metaverse is demonstrated through its applications in arts, medicine, and society within virtual environments (Wang et al., 2019; Aricò et al., 2020).

### 2.1 Metaverse art

Metaverse art pertains to art created and exhibited within virtual worlds or the metaverse, encompassing digital artwork, virtual reality installations, and other forms of artistic expression that exist within virtual environments. With the burgeoning popularity of virtual reality and immersive technologies, artists are exploring new ways to create and showcase their work within the metaverse, blurring the lines between physical and digital art spaces. The symbiotic relationship between art and technology is embodied by the metaverse, which originated from the field of art and in turn enriched it, creating a realm of virtual art that integrates reality and fantasy. Metaverse art encompasses various digital techniques such as virtual reality (VR), augmented reality (AR), mixed reality (MR), holographic projection, and non-fungible tokens (NFTs), drawing inspiration from the real world. The immersive exhibition “Qingming Shanghe Tu Immersive Art and Technology Exhibition,” held in Nanning in 2022, exemplifies Metaverse art. The exhibition employed holographic dynamic projection technology to animate a 1,000-year-old painting, enabling visitors to experience the painting’s surroundings through VR technology. Breaking away from the seriousness and distance of traditional art exhibitions, this exhibition endowed cultural relics with new vitality in the era of technology, providing visitors with an innovative and vivid esthetic experience. Due to its advantages in interaction and immersion, Metaverse art allows the general public to easily express themselves through art and deeply understand and participate in the works of Metaverse artists. Brain-computer interface technology has become a bridge for collaboration and communication between art creators and appreciators.

The intersection of Brain-Machine Interfaces and generative art has led to a burgeoning field in which artists relinquish their roles as creators to autonomous systems, such as computer programs and machines that take the lead or assist in the creation of a complete work of art (Hariz et al., 2016; Cheng et al., 2022). Through the autonomy required for generative art, Brain-Machine Interfaces are linked to this field. EEG signals collected from the brain are input into generative art devices, which are then activated. Due to the dynamic and unique nature of EEG signals, the output generated by the art device varies between individuals and at different times. Researchers have utilized NeuroSky MindWave headsets as signal collection tools, transmitting EEG data collected via Bluetooth to the ThinkGear Connector program on a computer. This program employs the User Datagram Protocol (UDP) to send data to the Unity engine, which then renders animations in real-time, projecting the results synchronously in front of the wearer of the headset. While rendering in real-time allows for partial autonomy, it also adheres to certain rules. Inspired by natural phenomena, researchers designed their rendering program based on the phyllotaxis of plants, utilizing a simple model of sunflower blossoms. They discovered that changing the angle of the flower could produce different pattern variations, and thus used angle as a variable to generate variable patterns. By extracting features from brainwave data, they mapped the degree of attention to different flower angles, and the degree of meditation to different flower colors. This unique approach visualizes the user’s level of calmness, and could be applied to meditation and art therapy (Lin et al., 2021). While this experiment was projected onto a two-dimensional plane and has yet to develop in the virtual space of the metaverse, using the metaverse as a canvas for generative art provides greater freedom and fewer economic costs than traditional two-or three-dimensional spaces. It offers artists a broader scope for inspiration and creativity (Lee et al., 2021). With the assistance of computers, the metaverse is highly efficient and simplifies the otherwise complex process of art creation (Zhao et al., 2022). The combination of Brain-Machine Interfaces and metaverse art makes the brain activity of individuals an integral component of the artwork. As brain activity varies between individuals and over time, it creates unique and dynamic art forms. By further understanding, the physiological reasons and symbolic meanings behind EEG signals, scientists and artists can enhance public understanding of brain function and develop more sophisticated art therapy methods through Brain-Machine Interfaces.

### 2.2 Metaverse medicine

Metaverse medicine utilizes VR, AR, and digital tech to transform healthcare. In medical education, immersive simulations enhance skills and readiness. Surgical simulations in the metaverse improve techniques and outcomes. It also supports collaborative research for complex cases, boosting diagnostic precision. In telemedicine, metaverse tech enables remote consultations and personalized therapy regimens, transcending geographical boundaries. This approach revolutionizes therapy and rehabilitation by replicating real-life challenges and monitoring progress interactively. Metaverse medicine leverages serious games in VR to enhance healthcare quality. These games tackle real-world challenges using innovative solutions. By simulating environments, they offer unique educational opportunities without safety or cost constraints. Popular metaverse games like Habbo Hotel, World of Warcraft, and Minecraft are examples.

In healthcare, serious games can improve physical and mental well-being through various activities like dance, exercise, self-management, and rehabilitation training. They also aid in diagnosing and treating mental illnesses. The field of serious games in healthcare is diverse, with ongoing exploration of brain-computer interface technology for therapeutic applications like Autism Spectrum Disorders (ASDs) (Stringfellow et al., 2024). ASD is a neurodevelopmental condition affecting communication, social interaction, and behavior (Hernandez et al., 2017). It varies in severity and manifests uniquely in individuals. Core features include social challenges, communication difficulties, repetitive behaviors, and sensory sensitivities. While there is no cure, early intervention and various therapies can improve outcomes. The current limitations in designing VR training systems for individuals with ASDs hinder assessing their neurological impact (Harfouche and Nakhle, 2020). Existing VR systems focus on behavioral training, lacking objective outcome measures. ASD individuals show mirror neuron dysregulation, with activity during self-movements but not when observing others. Normal individuals exhibit mirror neuron activation in both cases. Suppression of mu frequency (8–13 Hz) in the sensorimotor cortex indicates mirror neuron dysfunction level. Establishing behavioral improvement standards post-VR training is crucial. Comparing pre- and post-training EEG data can assess mirror neuron improvement (Oberman et al., 2005). Correlating behavioral outcomes with mirror neuron enhancement can indicate training effectiveness. Failure to observe improvements warrants further EEG research for ASD understanding and system design.

### 2.3 Social virtual reality

Social virtual reality (VR) entails the utilization of VR technology to construct interactive and shared experiences within a virtual environment (Huang et al., 2020). This innovation allows users to engage with one another as avatars in a simulated space, facilitating real-time communication, collaboration, and social activities. Social VR platforms encompass a plethora of features, including virtual gatherings, multiplayer games, virtual meetings, and collaborative workspaces (Kandalaft et al., 2013). These functionalities foster social connections and interactions in a digital realm. The potential of these platforms extends beyond mere entertainment; they have the ability to facilitate meaningful social experiences, transcending physical barriers. Social VR can be leveraged for socializing, entertainment, education, and professional networking, thereby introducing a novel dimension to individuals’ engagement and interaction within a virtual space (Redcay and Schilbach, 2019). By immersing users in captivating experiences, social virtual reality has the capacity to reshape the way people connect and communicate, bridging the gap between physical and digital social interactions.

In the realm of real-world social interactions, language serves as a fundamental tool, while facial expressions and body movements play crucial supportive roles (Redcay and Schilbach, 2019). Replicating users’ real-life expressions in virtual social worlds is a highly valuable endeavor. Taking Horizon Worlds as an example, as showcased in the pre-release concept video by Meta, users’ virtual avatars exhibit dynamic and authentic facial expressions, albeit limited to the upper body due to technological constraints. However, the current state of facial modeling and expression replication in Horizon Worlds falls short of expectations. To achieve real-time changes in facial expressions, meta employs emotion analysis of users’ voices, which is then mapped to corresponding facial changes. Nonetheless, due to limitations in facial modeling technology, these expression variations are restricted to combinations of specific facial sub-region movements such as raising eyebrows, opening and closing the mouth, and blinking (Brooks et al., 2021). As a result, the effects appear stiff and unnatural. To further enhance the simulation of facial expressions for virtual avatars, it is necessary not only to achieve esthetically pleasing facial modeling but also to employ methods that closely replicate natural expressions.

Facial expressions of humans can be classified into two types based on the methods used for emotion recognition: rule-based and machine learning-based. The first approach employs predefined rules and features to recognize and reproduce human facial expressions (Snoek et al., 2023). It relies on expert-defined rules and models to infer emotional states by calculating facial features such as eye shape, mouth movement, and eyebrow position, as well as vocal features like intonation and speech rate. Based on these emotional states, the system can generate corresponding facial expressions (Sowden et al., 2021). The advantage of this method is its interpretability, but it requires domain expertise and accurate rule definitions. In contrast, machine learning-based methods utilize machine learning algorithms to learn and recognize human facial expressions. This approach relies on a large amount of labeled data and employs feature extraction and classification algorithms to build emotion recognition models. Common features include facial expression features, vocal features, and language features. The model can predict emotional states based on input facial expressions, voice, or language and generate corresponding facial expressions. The advantage of this method is that it can automatically learn features and patterns, but it requires a significant amount of labeled data and training time. Both rule-based and machine learning-based methods can be utilized to reproduce human facial expressions. The choice of method depends on available resources, application scenarios, and accuracy requirements. As technology continues to advance, these methods are being further developed and improved to provide more accurate and natural reproduction of human facial expressions.

Integrating Brain-Machine Interface (BMI) into the metaverse may have profound implications for broader societal impacts, involving changes and challenges in various aspects (Sakellaridi et al., 2019). Firstly, this integration will fundamentally change the way humans communicate with each other. Through BMI technology, people can achieve direct interaction between the brain and computers or other devices, eliminating barriers of language and culture. This seamless form of communication may accelerate information transfer speed, enhance communication efficiency, but also bring risks of information overload and communication misunderstandings. Secondly, privacy issues will become a key challenge. BMI technology involves acquiring and processing individuals’ brain activity data, which contain highly private and sensitive information. In the metaverse, the use of BMI technology may expose individuals’ thoughts, emotions, etc., to unauthorized access or misuse, necessitating the establishment of strict privacy protection mechanisms and legal regulations to ensure the security and privacy rights of users are fully protected (Morsch et al., n.d.). Additionally, the metaverse integrated with BMI technology may give rise to new forms of social interaction. People can achieve deeper, more direct social connections by directly transmitting thoughts or emotions, potentially changing the traditional modes of social interaction. However, this also raises ethical and moral issues, such as distinguishing between real and virtual social interactions and maintaining human physical and mental health, posing challenges in various aspects (Bzdok and Dunbar, 2020). Overall, integrating BMI technology into the metaverse will bring many positive impacts to society, such as promoting cross-cultural communication, expanding communication channels, enhancing user experience, etc. (Sakellaridi et al., 2019). However, it is essential to address relevant privacy, ethical, and societal impacts seriously, ensuring that the application of technology complies with ethical standards, legal regulations, and maximizes benefits for the entire society.

## 3 Opportunities related to brain-machine interface technology

Brain-Machine Interface technology presents numerous opportunities in the present and future (Belardinelli et al., 2017). Firstly, it can enhance the quality of life for people with disabilities by helping those with limited mobility regain movement capabilities and assisting individuals who are deaf or blind to regain sensory perception. Secondly, BMI technology provides unprecedented opportunities for neuroscience research to explore brain structure and function, deepening our understanding of how the brain processes information and controls behavior. Additionally, BMI technology holds significant potential in virtual reality, augmented reality, and gaming, offering users a more immersive experience (Young et al., 2018). Finally, BMI technology can be used to develop new intelligent assistive systems, including brain signal-based human-machine interfaces and smart control systems. Overall, BMI technology will bring diverse opportunities and prospects in fields such as healthcare, scientific research, and technological innovation.

### 3.1 Neural security

In the current information society, the importance of neural security and network security issues is increasingly evident. Neural security involves safeguarding the confidentiality, integrity, and availability of neural devices and technologies to ensure that users’ privacy and data security are protected from malicious attacks. Meanwhile, as a result of the widespread application and rapid development of the internet, network security has emerged as an urgent challenge (Hie et al., 2018). Confidentiality is a key aspect of both neural and network security. In the realm of neural security, ensuring confidentiality entails preventing unauthorized access to users’ personal information and neural activity data. This involves implementing measures such as encrypted communication, identity authentication, and access control to ensure that data can only be accessed by authorized users. Similarly, in the context of network security, confidentiality concerns encompass data encryption, the use of secure transmission protocols, and the protection of network traffic to prevent the theft or unauthorized access of sensitive information. Integrity represents another crucial aspect of safeguarding neural devices and network security. In the domain of neural security, maintaining integrity necessitates preventing unauthorized modification and tampering with the settings or data of neural devices. This requires the implementation of tamper-resistant technical measures such as digital signatures, security authentication, and data integrity verification to ensure that device settings and data remain unaltered. Correspondingly, regarding network security, integrity issues involve preventing malicious software attacks, network packet tampering, and unauthorized modifications through measures like vulnerability patching, intrusion detection, and response actions to protect the integrity of systems and data (Yakneen et al., 2020). Furthermore, availability is also a significant consideration for both neural and network security. In the realm of neural security, ensuring availability requires that neural devices remain in normal working condition, free from the impact of denial-of-service attacks or other network intrusions. This may involve measures such as network traffic management, fault-tolerant mechanisms, and contingency plans to ensure that users can utilize neural devices without disruption. Similarly, in the context of network security, availability concerns encompass preventing distributed denial-of-service attacks, network fault recovery, and load balancing measures to ensure the continuity and reliability of network services.

### 3.2 Brain-hacking

The issue of brain-hacking refers to the illicit access, control, or manipulation of neural devices by attackers using technological means to obtain personal neural activity information or to influence individuals’ behavior (Dresler et al., 2019). Brain-hacking poses a threat to individuals’ neural mechanisms, neural computations, and free will by compromising the confidentiality, integrity, and availability of neural devices. Attackers may employ various methods to execute brain-hacking attacks, including but not limited to the following scenarios: neural data theft, whereby attackers unlawfully acquire personal neural activity information collected by neural devices (Karikari and Koshechkin, 2023). They may intercept, decode, or manipulate neural signals to obtain sensitive data such as electroencephalograms and brainwaves without authorization; intentional manipulation, where attackers alter the settings of neural devices or interfere with neural signals to manipulate users’ intentional activities. This may result in users making errors when controlling external applications or devices, or even being coerced into performing unwanted operations; neural device hijacking, where attackers gain complete control of devices and render them ineffective by intruding on the connection pathways or operating systems of neural devices. This could prevent users from using neural devices properly and may even cause damage to users’ neural systems. To counter brain-hacking attacks, a series of measures need to be implemented to safeguard the security of neural devices. These include encryption and authentication, which involve employing encryption technology to protect neural signals, ensuring that data is not stolen or tampered with during transmission and storage. Additionally, the use of authentication mechanisms ensures that only authorized users can access and operate neural devices.

### 3.3 Neuroethics

Neuroethical concerns encompass an emerging interdisciplinary field that investigates the relationship between neuroscience and ethical principles (Cometa et al., 2022). It delves into the ethical issues arising from the development of neuroscience and neurotechnologies, offering corresponding moral guidelines and norms. This field holds significant importance in exploring and resolving increasingly complex ethical quandaries. In the realm of brain research, neuroscience reveals the structure and functionality of the brain; however, it may also encroach upon personal privacy. Consequently, striking a balance between scientific progress and the need to protect individual privacy becomes a pivotal ethical concern. Furthermore, advancements in neurotechnology enable enhancements of cognitive abilities, learning capabilities, and motor skills (Borton et al., 2020). Yet, if only a select few can avail themselves of neural augmentation, might it exacerbate social inequality? Ensuring adherence to principles of fairness and justice in the application of neural enhancement technologies thus becomes a crucial ethical consideration. Brain-computer interface (BMI) technology allows individuals to directly interact with computer systems using brain signals, but it also raises concerns regarding personal identity and data privacy. Safeguarding personal BMI data to prevent misuse and potential infringement upon individual rights demands serious ethical deliberation. Additionally, the progress in neurotechnology enables interventions in the human brain. However, intervening in an individual’s brain raises questions of autonomy and individual agency. Thus, determining appropriate ethical standards and decision-making processes in neurointerventions, while ensuring voluntariness and individual choice, presents a significant ethical challenge.

### 3.4 Commercialization in neural technology

The issue of commercialization in neural technology has arisen as a result of rapid developments in neuroscience and neurotechnology, leading some companies to integrate these technologies within their commercial products and services, such as brain-computer interface devices and neural enhancement technologies (Himmelfarb et al., 2020). This has raised concerns regarding personal data privacy and security. The collection, storage, and use of brain signals and neural data may lead to potential exposure and misuse of personal information, warranting the establishment of appropriate privacy protection and data security mechanisms. Additionally, the commercialization of neural technology has sparked questions about fairness and moral competition. If certain individuals can gain cognitive or motor advantages through neural enhancement technologies, could this exacerbate social inequality? It is imperative to consider how to ensure that the commercialization of neural technology does not further divide society but instead promotes wider welfare and benefits. Moreover, the commercialization of neural technology presents challenges related to consumer rights and informed consent. Ensuring consumers fully comprehend associated risks and effects and can make independent, informed decisions is an essential ethical issue throughout the development and sales process of neural technology products and services. Furthermore, regulation and standardization of neural technology products are critical issues that urgently need resolution to preserve consumer rights and public interests. Currently, commercial brain-computer interface products have been restricted to researchers and technology enthusiasts, with relatively high prices further hindering their popularity and dissemination (Young et al., 2018).

### 3.5 Ethical and privacy issues

Ensuring the protection of user privacy and data security is paramount when it comes to Brain-Machine Interface (BMI) technology. Given that BMI technology involves the acquisition and processing of highly sensitive biological signals from individuals’ brains, safeguarding data security and privacy becomes imperative. It is essential to implement robust measures to secure this sensitive data, prevent unauthorized access or misuse, and adhere to strict compliance with pertinent regulations and ethical guidelines. Moreover, establishing comprehensive protocols for the responsible and ethical use of BMI data is crucial. This includes defining clear guidelines for data collection, storage, access control, and encryption methods to prevent any breaches or unauthorized disclosures. By implementing stringent data protection measures, organizations can instill trust among users and ensure that their personal information remains confidential and secure. Furthermore, promoting transparency in how BMI data is collected, used, and shared is essential for fostering user confidence.

## 4 Case studies of brain-machine interface technology in different fields

### 4.1 Neuroscience research

In neuroscience, BMI technology records brain signals through implanted electrode arrays or sensors, aiding researchers in understanding the workings and cognitive functions of the brain (Eles et al., 2018). This technology enables researchers to observe how information is transmitted between different regions of the brain and investigate the relationship between specific behaviors or cognitive tasks and neural activity. BMI technology provides scientists with vital tools and insights to better understand how the brain processes information, makes decisions, and performs control functions.

### 4.2 Rehabilitation medicine

Brain-Machine Interface technology plays a crucial role in rehabilitation medicine, providing essential means for people with disabilities (Bonizzato et al., 2018). Through brain-controlled prosthetic technology, amputees can regain limb functionality and achieve precise control of prosthetics. This technology not only helps patients regain autonomy and independence but also promotes innovation and development in prosthetic technology. By using BMI technology, rehabilitation medicine can offer more personalized and effective rehabilitation programs for people with disabilities, improving their quality of life.

### 4.3 Treatment of language disorders

Some research teams utilize BMI technology to treat patients with language disorders, such as aphasia patients (Young et al., 2018). By monitoring brain activity and interacting with computers, patients can overcome language communication barriers by inputting text or speech through thoughts. This technology offers new possibilities for language rehabilitation, helping patients reestablish language and communication abilities. The application of BMI technology makes language disorder treatment more personalized and effective, bringing new hope to the field of language rehabilitation.

### 4.4 Virtual reality and gaming

In the fields of virtual reality and gaming, BMI technology is used to enhance user experience and interactivity (Filipp et al., 2019). By monitoring brain signals and applying them to virtual environments or games, players can control game characters or influence the development of the virtual world through brain activity. This technology creates a more immersive and personalized user experience, driving innovation and development in virtual reality and gaming technology. The application of BMI technology brings new possibilities to the virtual reality and gaming industry, expanding the boundaries of user experience.

### 4.5 Military applications

In the military domain, BMI technology is considered a significant technology for future warfare with strategic importance (Latheef, 2023). Military departments are researching how to use BMI technology to help soldiers control complex equipment and systems, enhancing combat effectiveness and reaction speed. Furthermore, some defense departments are exploring the development of brain-controlled drones or weapon systems to strengthen military capabilities and battlefield advantages. These applications provide new possibilities for the development of military technology and changes in warfare strategies.

## 5 Challenges of brain-machine interface technology

### 5.1 Signal noise

Signal noise is a common challenge in BMI technology. Brain signals are often weak and susceptible to external interference such as muscle movements, electromagnetic interference, etc., leading to a decrease in signal quality and accuracy (Ruda et al., 2020). The presence of signal noise affects the stability and performance of BMI systems, limiting the precision and reliability of brain-controlled devices. Addressing signal noise issues requires the implementation of signal processing algorithms, sensor design, and interference suppression techniques to improve signal quality and accuracy.

### 5.2 Interpretational accuracy

Another significant challenge is the accurate interpretation of brain signals by BMI systems. Brain signals are typically complex and variable, with significant differences between individuals, and the mapping between brain activity and specific behaviors or intentions is not entirely clear (Broccard et al., 2014). Therefore, accurately interpreting brain signals and translating them into meaningful commands or control signals is a challenging problem. Current interpretation algorithms and models need continuous improvement and optimization to enhance the accuracy and reliability of BMI systems.

### 5.3 Limitations of non-invasive methods

Currently, most BMI technologies utilize invasive methods such as implanting electrodes or sensors to record brain signals. While invasive methods can provide high signal-to-noise ratios and higher spatial resolution, their limitations are evident (Wang et al., 2019; Aricò et al., 2020). Invasive surgeries pose risks of infection and rejection, while the long-term stability and durability of implants face challenges. To overcome these issues, researchers are working on developing non-invasive BMI technologies, such as those based on functional magnetic resonance imaging (fMRI) or electroencephalography (EEG). However, non-invasive methods typically have lower signal quality and spatial resolution compared to invasive methods, highlighting the need to balance the advantages and disadvantages of invasive and non-invasive methods.

## 6 Advancements in signal processing for brain-machine interface technology

### 6.1 High-resolution signal acquisition technology

High-resolution acquisition of brain signals has been a recent research focus. Using advanced electroencephalography (EEG) and functional magnetic resonance imaging (fMRI) devices allows for finer information on brain activity, thereby improving signal quality and accuracy.

### 6.2 Real-time signal processing and decoding algorithms

Researchers are dedicated to developing faster and more accurate signal decoding algorithms for the real-time signal processing demands of brain-machine interfaces. Machine learning-based methods, including Support Vector Machines (SVM), deep learning models such as Convolutional Neural Networks (CNN), are widely applied for pattern recognition and decoding of brain signals to accurately capture user intent.

### 6.3 Multimodal signal fusion technology

Recent studies indicate that fusing various types of brain signals can comprehensively reflect brain activity and enhance the accuracy of signal processing and decoding. Therefore, multimodal signal fusion technology is receiving increasing attention and application in BMI technology.

## 7 Conclusion

As immersion and functional needs in the metaverse grow, BMI technology has the potential to revolutionize metaverse interaction. BMI enable direct information acquisition from the human brain, offering a natural, intuitive way to interact with the metaverse. Users can control virtual elements through thoughts, enhancing freedom and personalization. Future focus will likely increase on BMI applications in the metaverse, improving performance, reliability, and addressing security and ethical concerns. Collaboration among governments, academia, and businesses is crucial to establish regulations ensuring ethical BMI use, maximizing benefits for society.

## Author contributions

YL: Investigation, Writing – original draft. RL: Investigation, Writing – original draft. JG: Investigation, Validation, Writing – review & editing. YW: Conceptualization, Supervision, Writing – original draft, Writing – review & editing.

## References

[ref1] Aricò P. Sciaraffa N. Babiloni F. (2020). Brain-machine interfaces: toward a daily life employment. Brain Sci. 10:157. doi: 10.3390/brainsci10030157, PMID: 32182818 PMC7139579

[ref2] Baumeister A. A. (2000). The Tulane electrical brain stimulation program a historical case study in medical ethics. J. Hist. Neurosci. 9, 262–278. doi: 10.1076/jhin.9.3.262.1787, PMID: 11232368

[ref3] Belardinelli P. Laer L. Ortiz E. Braun C. Gharabaghi A. (2017). Plasticity of premotor cortico-muscular coherence in severely impaired stroke patients with hand paralysis. Neuroimage Clin. 14, 726–733. doi: 10.1016/j.nicl.2017.03.005, PMID: 28409112 PMC5379882

[ref4] Bhugaonkar K. Bhugaonkar R. Masne N. (2022). The trend of metaverse and augmented & virtual reality extending to the healthcare system. Cureus 14:e29071. doi: 10.7759/cureus.29071, PMID: 36258985 PMC9559180

[ref5] Bonizzato M. Pidpruzhnykova G. DiGiovanna J. Shkorbatova P. Pavlova N. Micera S.. (2018). Brain-controlled modulation of spinal circuits improves recovery from spinal cord injury. Nat. Commun. 9:3015. doi: 10.1038/s41467-018-05282-6, PMID: 30068906 PMC6070513

[ref6] Borton D. A. Dawes H. E. Worrell G. A. Starr P. A. Denison T. J. (2020). Developing collaborative platforms to advance neurotechnology and its translation. Neuron 108, 286–301. doi: 10.1016/j.neuron.2020.10.001, PMID: 33120024 PMC7610607

[ref7] Broccard F. D. Mullen T. Chi Y. M. Peterson D. Iversen J. R. Arnold M.. (2014). Closed-loop brain-machine-body interfaces for noninvasive rehabilitation of movement disorders. Ann. Biomed. Eng. 42, 1573–1593. doi: 10.1007/s10439-014-1032-6, PMID: 24833254 PMC4099421

[ref8] Brooks J. Teng S. K. Wen J. Nith R. (2021). “Stereo-smell via electrical trigeminal stimulation” in Proceedings of the 2021 CHI Conference on Human Factors in Computing Systems. Yokohama, 1.

[ref9] Bzdok D. Dunbar R. I. M. (2020). The neurobiology of social distance. Trends Cogn. Sci. 24, 717–733. doi: 10.1016/j.tics.2020.05.016, PMID: 32561254 PMC7266757

[ref10] Cheng Q. M. (2022). Structural forms of metaverse and the characteristics of metaverse art. J. Zhejiang Shuren Univ. 22:78,

[ref11] Cometa A. Falasconi A. Biasizzo M. Carpaneto J. Horn A. Mazzoni A.. (2022). Clinical neuroscience and neurotechnology: an amazing symbiosis. iScience 25:105124. doi: 10.1016/j.isci.2022.105124, PMID: 36193050 PMC9526189

[ref12] Dresler M. Sandberg A. Bublitz C. Ohla K. Trenado C. Mroczko-Wąsowicz A.. (2019). Hacking the brain: dimensions of cognitive enhancement. ACS Chem. Neurosci. 10, 1137–1148. doi: 10.1021/acschemneuro.8b00571, PMID: 30550256 PMC6429408

[ref13] Eles J. R. Vazquez A. L. Kozai T. D. Y. Cui X. T. (2018). In vivo imaging of neuronal calcium during electrode implantation: spatial and temporal mapping of damage and recovery. Biomaterials 174, 79–94. doi: 10.1016/j.biomaterials.2018.04.043, PMID: 29783119 PMC5987772

[ref14] Filipp M. E. Travis B. J. Henry S. S. Idzikowski E. C. Magnuson S. A. Loh M. Y.. (2019). Differences in neuroplasticity after spinal cord injury in varying animal models and humans. Neural Regen. Res. 14, 7–19. doi: 10.4103/1673-5374.243694, PMID: 30531063 PMC6263009

[ref15] Harfouche A. L. Nakhle F. (2020). Creating bioethics distance learning through virtual reality. Trends Biotechnol. 38, 1187–1192. doi: 10.1016/j.tibtech.2020.05.005, PMID: 32446631 PMC7211661

[ref16] Hariz M. Blomstedt P. Zrinzo L. (2016). Deep brain stimulation between 1947 and 1987: the untold story. Neurosurg. Focus. 29:e1. doi: 10.3171/2010.4.FOCUS10106 20672911

[ref17] Hernandez L. M. Krasileva K. Green S. A. Sherman L. E. Ponting C. McCarron R.. (2017). Additive effects of oxytocin receptor gene polymorphisms on reward circuitry in youth with autism. Mol. Psychiatry 22, 1134–1139. doi: 10.1038/mp.2016.209, PMID: 27843152 PMC5991611

[ref18] Hie B. Cho H. Berger B. (2018). Realizing private and practical pharmacological collaboration. Science 362, 347–350. doi: 10.1126/science.aat4807, PMID: 30337410 PMC6519716

[ref19] Himmelfarb J. Vanholder R. Mehrotra R. Tonelli M. (2020). The current and future landscape of dialysis. Nat. Rev. Nephrol. 16, 573–585. doi: 10.1038/s41581-020-0315-4, PMID: 32733095 PMC7391926

[ref20] Huang K. H. Rupprecht P. Frank T. Kawakami K. Bouwmeester T. Friedrich R. W. (2020). A virtual reality system to analyze neural activity and behavior in adult zebrafish. Nat. Methods 17, 343–351. doi: 10.1038/s41592-020-0759-2, PMID: 32123394 PMC7100911

[ref21] Kandalaft M. R. Didehbani N. Krawczyk D. C. Allen T. T. Chapman S. B. (2013). Virtual reality social cognition training for young adults with high-functioning autism. J. Autism Dev. Disord. 43, 34–44. doi: 10.1007/s10803-012-1544-6, PMID: 22570145 PMC3536992

[ref22] Karikari E. Koshechkin K. A. (2023). Review on brain-computer interface technologies in healthcare. Biophys. Rev. 15, 1351–1358. doi: 10.1007/s12551-023-01138-6, PMID: 37974976 PMC10643750

[ref23] Kuruvilla A. Flink R. (2003). Intraoperative electrocorticography in epilepsy surgery: useful or not? Seizure 12, 577–584. doi: 10.1016/S1059-1311(03)00095-5 14630497

[ref24] Kye B. Han N. Kim E. Park Y. Jo S. (2021). Educational applications of metaverse: possibilities and limitations. J Educ Eval Health Prof. 18:32. doi: 10.3352/jeehp.2021.18.32, PMID: 34897242 PMC8737403

[ref25] Latheef S. (2023). Brain to brain interfaces (BBIs) in future military operations; blurring the boundaries of individual responsibility. Monash Bioeth. Rev. 41, 49–66. doi: 10.1007/s40592-022-00171-7, PMID: 36550229

[ref26] Lee LH Lin ZJ Hu R Gong Z Abhishek Kumar Li T. (2021). When creators meet the metaverse: a survey on computational arts. ArXiv [Preprint]. doi: 10.48550/arXiv.2111.13486

[ref27] Lin C. T. Rajapakse R. P. C. J. Tokuyama Y. (2021). Development of EEG data-driven generative art application for real-time and dynamic interaction. J. Robot. Network. Artif. Life 8:117. doi: 10.2991/jrnal.k.210713.010

[ref28] Morsch R. Landgraeber S. Strauss D. J. (n.d.). Neue perspektiven in der orthopädie Entwicklungen durch Neurotechnologie und Metaversum [New perspectives in orthopedics: Developments through neurotechnology and metaverse]. Orthopadie (Heidelb). 2023 Orthopadie 52, 547–551. doi: 10.1007/s00132-023-04400-7 37289216

[ref29] Oberman L. M. Hubbard E. M. McCleery J. P. Altschuler E. L. Ramachandran V. S. Pineda J. A. (2005). EEG evidence for mirror neuron dysfunction in autism spectrum disorders. Cogn. Brain Res. 24, 190–198. doi: 10.1016/j.cogbrainres.2005.01.014, PMID: 15993757

[ref30] Palmini A. (2006). The concept of the epileptogenic zone: a modern look at Penfield and Jasper's views on the role of interictal spikes. Epileptic Disord. 8, S10–S15. doi: 10.1684/j.1950-6945.2006.tb00205.x 17012068

[ref31] Redcay E. Schilbach L. (2019). Using second-person neuroscience to elucidate the mechanisms of social interaction. Nat. Rev. Neurosci. 20, 495–505. doi: 10.1038/s41583-019-0179-4 31138910 PMC6997943

[ref32] Ritterbusch G. D. Teichmann M. R. (2023). Defining the metaverse: a systematic literature review. IEEE Access. 11, 12368–12377. doi: 10.1109/ACCESS.2023.3241809

[ref33] Ruda K. Zylberberg J. Field G. D. (2020). Ignoring correlated activity causes a failure of retinal population codes. Nat. Commun. 11:4605. doi: 10.1038/s41467-020-18436-2, PMID: 32929073 PMC7490269

[ref34] Sakellaridi S. Christopoulos V. N. Aflalo T. Pejsa K. W. Rosario E. R. Ouellette D.. (2019). Intrinsic variable learning for brain-machine interface control by human anterior intraparietal cortex. Neuron 102, 694–705.e3. doi: 10.1016/j.neuron.2019.02.012, PMID: 30853300 PMC6922088

[ref35] Snoek L. Jack R. E. Schyns P. G. Garrod O. G. B. Mittenbühler M. Chen C.. (2023). Testing, explaining, and exploring models of facial expressions of emotions. Sci. Adv. 9:eabq8421. doi: 10.1126/sciadv.abq8421 36763663 PMC9916981

[ref36] Sowden S. Schuster B. A. Keating C. T. Fraser D. S. Cook J. L. (2021). The role of movement kinematics in facial emotion expression production and recognition. Emotion 21, 1041–1061. doi: 10.1037/emo0000835, PMID: 33661668 PMC8582590

[ref37] Stringfellow MK Fields NL Lee K Anderson KA Brokaw E. Healthy aging and older adults with autism: a scoping review. Gerontologist (2024):gnae026. doi: 10.1093/geront/gnae026 (Epub ahead of print). 38520290

[ref38] Wang Z. H. Yu Y. Xu M. Liu Y. Yin E. Zhou Z. (2019). Towards a hybrid BMI gaming paradigm based on motor imagery and SSVEP. Int. J. Hum. Comput. Interact. 35, 197–205. doi: 10.1080/10447318.2018.1445068

[ref39] Wang W. Zhou F. Wan Y. Ning H. (2022). A survey of metaverse technology. Chin. J. Eng. 44, 744–756,

[ref40] Yakneen S. Waszak S. M. PCAWG Technical Working Group Gertz M. Korbel J. O. PCAWG Consortium (2020). Butler enables rapid cloud-based analysis of thousands of human genomes. Nat. Biotechnol. 38, 288–292. doi: 10.1038/s41587-019-0360-3 32024987 PMC7062635

[ref41] Young A. T. Cornwell N. Daniele M. A. (2018). Neuro-nano interfaces: utilizing nano-coatings and nanoparticles to enable next-generation electrophysiological recording, neural stimulation, and biochemical modulation. Adv. Funct. Mater. 28:1700239. doi: 10.1002/adfm.201700239, PMID: 33867903 PMC8049593

[ref42] Zhao Y. Jiang J. Chen Y. Liu T. Yang T. Xue X.. (2022). Metaverse: perspectives from graphics, interactions and visualization. Visual Info. 6, 56–67. doi: 10.1016/j.visinf.2022.03.002

